# Clonal diversity of carbapenemase-producing *Pseudomonas aeruginosa* isolated from clinical samples in a third level hospital in Peru

**DOI:** 10.17843/rpmesp.2025.421.13818

**Published:** 2025-02-17

**Authors:** Gina Salvador-Lujan, Liz Erika Cruz-Pio, Hedersson Calla, Damaris Rivera-Asencios, Luis Solís-Cayo, Ruth García-de-la-Guarda

**Affiliations:** 1 Microbiology Laboratory of the Central Military Hospital “Crl. Luis Arias Schreiber”, Lima, Peru. Microbiology Laboratory of the Central Military Hospital “Crl. Luis Arias Schreiber” Lima Perú; 2 Microbial Ecology Laboratory, Faculty of Biological Sciences, Universidad Nacional Mayor de San Marcos, Lima, Peru. Microbial Ecology Laboratory Faculty of Biological Sciences Universidad Nacional Mayor de San Marcos Lima Perú; 3 Laboratory of Molecular Microbiology and Biotechnology, Faculty of Biological Sciences, Universidad Nacional Mayor de San Marcos, Lima, Peru. Laboratory of Molecular Microbiology and Biotechnology Faculty of Biological Sciences Universidad Nacional Mayor de San Marcos Lima Perú

**Keywords:** Pseudomonas aeruginosa, Metallo-β-lactamases, Molecular Typing, Carbapenemases

## Abstract

*Pseudomonas aeruginosa* is an opportunistic pathogen associated with health care infections, it has high levels of antimicrobial resistance and is associated with hospital outbreaks. Early outbreak detection is a usual problem in hospitals, therefore, this study aimed to assess the clonal relationship of carbapenemase-producing *P. aeruginosa* in a tertiary hospital in Lima, Peru. Twenty-four metallo β-lactamase-producing *P. aeruginosa* strains isolated from hospitalized patients were collected. The clonal relation was determined using the REP-PCR technique. REP-PCR band profiles were normalized, analyzed and combined using BioNumerics version 7.6 software. Molecular identification showed 19 different profiles and four clonal groups. We determined polyclonality among isolates. We did not find clonal dissemination among the metallo-β-lactamase-producing P. aeruginosa strains circulating in the hospital.

## INTRODUCTION

*Pseudomonas aeruginosa* is considered an opportunistic pathogen of great clinical relevance due to the high morbidity and mortality associated with healthcare-associated infections ^1^^,^^2^. In 2020, the Centers for Disease Control and Prevention (CDC) reported 28,800 cases and 2,500 deaths from multidrug-resistant (MDR) *P. aeruginosa* in hospitalized patients, figures that increased during the COVID-19 pandemic ^3^.

The treatment of *P. aeruginosa* infections is increasingly limited due to its high capacity to develop resistance to antimicrobials through mechanisms such as constitutive expression of *Pseudomonas*-derived cephalosporinases (PDC), efflux pump modifications, low outer membrane permeability, beta-lactamase production, and adaptive resistance to stress conditions, making it an important public health problem ^1^^,^^2^^,^^4^.

Resistance to carbapenem antibiotics (the last resort for MDR bacterial infections) in *P. aeruginosa* is mainly due to the decrease in OprD, overexpression of efflux pumps, overproduction of PDC, and/or a combination of these mechanisms. However, carbapenemases encoded in plasmids and integrons acquired by horizontal transfer are highly relevant, particularly metallo-β-lactamases (MBL) ^2^^,^^4^. MBLs have several variants that cause nosocomial spread of high-risk clones and MDR strains that are extremely resistant (XDR), pan-resistant (PDR), or difficult to treat (DTR). Therefore, it is important to monitor the spread of clones for early detection and to prevent outbreaks ^1^^,^^4^. Among the reference techniques used for molecular typing is pulsed-field gel electrophoresis (PFGE) due to its high discriminatory power. However, its limitations include the high cost of the equipment, its laborious nature, the difficulty of differentiating pulsotypes with nearby DNA bands, and the time required to analyze the pulsotypes ^5^^,^^6^.

Repeated DNA sequences (REP-PCR) are a different type of techniques, which are inexpensive, less labor-intensive, fast, and tend to generate band patterns that are relatively easier to interpret than those obtained by PFGE, although the discriminatory power of this PCR technique may be lower than that obtained by PFGE ^5^. The REP-PCR (Repetitive Extragenic Palindromic) technique amplifies repeated sequences with palindromic elements between 35-40 bp, giving rise to a wide variability of DNA band profiles depending on the number of repetitive sequences and the distance between them, which is why it is successfully used for epidemiological and taxonomic purposes and marketed in semi-automated formats such as Diversilab (bioMérieux) ^7^.

In 2016, at the Central Military Hospital, 24 strains of *P. aeruginosa* that were highly resistant to antibiotics and produced MBL were isolated and identified from different hospital wards ^8^. This study aimed to investigate the genetic relationship (clonality) between MBL strains of *P. aeruginosa* using the REP-PCR technique.

**KEY MESSAGES**

Motivation for the study. The isolation of carbapenemase-producing *Pseudomonas aeruginosa* in different wards of a tertiary care hospital prompted the identification of the clonality of the isolates and to determine whether they corresponded to an intrahospital outbreak.

Main findings. The REP-PCR technique grouped the 24 strains of metallo-β-lactamase-producing *P. aeruginosa* isolated from patients in different hospital wards into 19 profiles. The greatest clonal diversity was found in the medical ward.

Public health implications. Molecular typing by REP-PCR could be a practical and rapid alternative for the surveillance and control of hospital outbreaks.

## THE STUDY

A descriptive cross-sectional study was conducted. We used twenty-four strains of carbapenem-resistant *P. aeruginosa* isolated from respiratory secretions, wounds, urine, and blood between January and September 2016. Susceptibility to antimicrobials: imipenem, meropenem, ceftazidime, cefepime, piperacillin tazobactam, ciprofloxacin, gentamicin, and amikacin was determined by disk diffusion according to the Clinical and Laboratory Standards Institute (CLSI) ^9^ and colistin by broth disk elution (CLSI). The definitions of MDR and XDR were based on Magiorakos *et al*. ^10^ and those of DTR on Kadri *et al*. ^11^.

The strains were reactivated and seeded in Luria-Bertani broth (Difco, Le Pont de Claix, France) pH 7.0 and incubated at 37 °C for 12-14 hours. The Wizard® Genomic DNA Purification Kit (Promega, Madison, Wisconsin, USA) was used to extract genomic DNA following the manufacturer’s instructions. The extracted and purified genomic DNA was quantified using a NanoDrop One (ThermoFisher Scientific, Madison, USA).

The Applied Biosystems ProFlex PCR System (ThermoFisher Scientific, Madison, USA) thermocycler was used for molecular analysis by REP-PCR, along with the REP-1-F (5’-III GCGCCGICATCAGGC-3’) and REP-2-R (5’-ACGTCTTATCAGGCCTAC-3') primers previously described by Versalovic *et al*. ^12^ with the following modifications in the amplification conditions: an initial denaturation (95 °C for 2 minutes), followed by 30 cycles of denaturation (95 °C for 30 seconds), hybridization (60 °C for 45 seconds), extension (70 °C for 1 minute), and a final extension cycle at 70 °C for 10 minutes. Each PCR reaction was carried out in a total volume of 25 µL, using KOD Hot Start DNA Polymerase (Novagen Millipore, Billerica, MA USA) and 1 µL of purified genomic DNA as template. Simultaneously, molecular biology-grade water was used as a negative control, and *P. aeruginosa* ATCC 27853 and *Escherichia coli* ATCC 25922 were used as amplification controls.

The amplification products were separated on 1.5% (w/v) agarose gel (Sigma-Aldrich, St. Louis, MO, USA). For electrophoresis, 50 mM TAE buffer (50 mM Tris acetate EDTA, pH 8.5) was used at 50 V for 2.5 hours. The size of the fragments was estimated by comparison with the GeneRuler 1 kb DNA Ladder molecular weight marker (ThermoFisher Scientific, Madison, USA) and the bands were visualized with GelRed® Nucleic Acid Gel Stain (Biotium, San Francisco Bay Area, USA) at 20X and digitalized with a documentation system.

The REP-PCR band profiles were normalized, analyzed, and combined using BioNumerics version 7.6 (Applied Maths, Kortrijk, Belgium). The discrimination threshold was determined according to the methodology of Rodas *et al*. ^13^^)^ and Cruz-Pio *et al*^14^.

This research was approved by the Ethics Committee of the Central Military Hospital (Official Letters: No. 285AA-11/a/01.01 and No. 1193AA-11/8/HMC/DADCI). The identifying data of the isolated strains were treated confidentially.

## FINDINGS

The samples of 54.2% of MBL-producing *P. aeruginosa* were collected from respiratory secretions, 29.2% from wounds, 12.5% from urine, and 4.1% from blood. The *bla*_IMP_ (95.8%) and *bla*_VIM_ (4.2%) genes were identified. According to the hospital unit, they were distributed as follows: 79.2% in medicine, 12.5% in the intensive care unit (ICU), and 4.2% in surgery and pulmonology ^8^ (Table 1).

**Table 1:** Metallo-β-lactamases-producing *Pseudomonas aeruginosa* and their relationship with samples from hospitalized patients.

Code	Strain			Patient data		Hospital unit
	Date of isolation	Sample	***bla* gene**	Sex	Age	
334	04-29-2016	SR	IMP	M	82	MED
414	16-15-2016	SR	IMP	M	52	MED
751	09-08-2016	SR	IMP	M	75	MED
809	09-23-2016	SR	IMP	M	72	MED
28	01-25-2016	SR	IMP	M	69	MED
383	06-04-2016	SR	IMP	M	97	MED
869	10-05-2016	SR	IMP	M	32	MED
814	06-25-2016	SR	IMP	F	59	MED
925	10-17-2016	SR	IMP	M	76	MED
408	06-14-2016	H	IMP	M	64	MED
707	09-02-2016	H	IMP	M	64	MED
147	02-12-2016	H	IMP	M	85	MED
862	10-01-2016	H	IMP	M	54	MED
912	10-13-2016	H	IMP	F	55	MED
47	01-30-2016	H	IMP	M	26	MED
792	09-20-2016	H	IMP	M	66	MED
369	05-06-2016	O	IMP	F	82	MED
347	03-06-2016	O	VIM	M	63	MED
382	09-08-2016	HEMO	IMP	M	37	MED
539	07-24-2016	SR	IMP	M	89	UCI
769	07-14-2016	SR	IMP	M	56	UCI
778	07-15-2016	SR	IMP	M	87	UCI
333	04-29-2016	SR	IMP	M	20	NEU
947	10-24-2016	O	IMP	M	90	SUR

MED: medicine ward; ICU: intensive care unit; PUL: pulmonology; SUR: surgery; W: wound; RS: respiratory secretion; U: urine; HEMO: blood; F: female; M: male; blaIMP: Imipenemase gene; blaVIM: Verona Integron-encoded Metallo β-lactamase gene.

**Figure 1:**
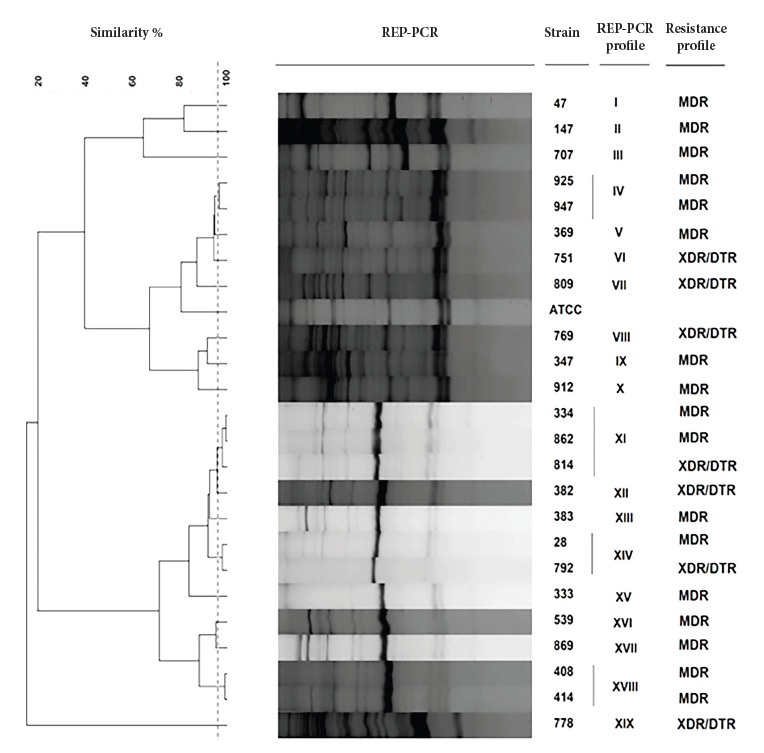
Dendrogram showing the clonal relationship of 24 strains of *Pseudomonas aeruginosa* producing metallo-β-lactamases, using Pearson’s coefficient of co-phenetic correlation and the UPGMA (Unweighted-Pair Group Method with Arithmetic Mean) grouping method. The dotted line represents 96% similarity.

MDR: multidrug resistant; XDR: extremely drug resistant; DTR: difficult to treat; ATCC: American Type Culture Collection

All samples were resistant to meropenem, imipenem, and ceftazidime, 95.8% to cefepime, 87.5% to gentamicin and ciprofloxacin, 83.3% to amikacin, 33.3% to piperacillin tazobactam, and 12.5% to aztreonam ^8^. All were susceptible to colistin and, according to the resistance profile, 70.8% (17/24) were MDR and 29.2% (7/24) were XDR/DTR. No PDR profiles were detected (Figure 1).

After typing, the 24 strains of *P. aeruginosa* MBL were grouped into 19 REP-PCR profiles with a similarity level of 96% and a Pearson correlation coefficient of 90% (optimized at 1%, tolerance at 2.5%), meaning that these 19 profiles of *P. aeruginosa* MBL profiles are genetically distinct and will henceforth be considered as distinct strains (Figure 1). The reproducibility rate of the obtained profiles was 98% and was the result of the average similarity value of three strains analyzed in duplicate.

We found that 78.9% (15/19) of the REP-PCR profiles were represented by a single strain and presented unique REP-PCR profiles, i.e., with no genetic relationship between them; of these, 73.3% (11/15) corresponded to the medicine ward. Profiles VIII, XVI, and XIX were found in the ICU, representing 15.8% of the unique profiles. Profile XV was found in the pulmonology ward, and profile IV was found in the surgery ward, representing 5.3% in both cases.

Four clonal groups were found: profile IV (strains 925 and 947) in the medicine and surgery wards; as well as profile XI (strains 334, 862, and 814), profile XIV (strains 28 and 792); and profile XVIII (strains 408 and 414). The latter three clonal groups were found in the medicine ward.

According to the resistance profile, 82.3% (17/24) of MDR and 71.4% (5/7) of DTR were isolated in the medicine ward, while 28.6% (2/7) of DTR strains were isolated in the ICU. The unit with the highest number of strains and diversity of profiles was medicine, with 11 unique profiles and 3 clonal groups.

## DISCUSSION

In this article, we report the high clonal diversity of MBL *P. aeruginosa* with MDR and XDR/DTR resistance profiles circulating mainly in the medical unit of a tertiary care hospital in Lima.

Carbapenem-resistant *P. aeruginosa* is one of the main microorganisms causing healthcare-associated infections. For this reason, the World Health Organization continues to include it on its list of bacterial pathogens that are a high priority for research, development, and the search for new strategies for infection prevention and control ^15^. Early detection of carbapenemase-producing *P. aeruginosa* in colonized or infected patients is of utmost importance in healthcare facilities to prevent its spread and hospital outbreaks.

Globally, carbapenem-resistant *P. aeruginosa* infections have been reported in hospitalized patients, causing outbreaks in different hospital units, mainly ICUs ^16^^,^^17^. During 2016, the Central Military Hospital reported an increase in MBL-resistant *P. aeruginosa* isolates, identifying the *bla*_IMP_ and *bla*_VIM_ genes ^8^. We found that 69.2% of respiratory secretions were isolated from the medicine ward, findings consistent with those by Vega *et al*. ^2^, who reported that 51.3% of their MBL *P. aeruginosa* isolates were from respiratory infections in an academic healthcare center in Miami, Florida.

Currently, the spread of high-risk clones of MDR/XDR *P. aeruginosa* is a public health problem, jeopardizing current antimicrobial therapy and increasing morbidity and mortality ^18^. In Spain, Sastre-Femenia *et al*. ^19^^)^ reported a decrease in XDR and DTR resistance profiles of *P. aeruginosa* and a slight decrease in the prevalence of extended-spectrum beta-lactamases and carbapenemases in 2022; however, there was an increase in XDR strains producing carbapenemases associated with the high-risk hypervirulent ST235 clone. In our study, 70.8% of MBL *P. aeruginosa* were MDR and 29% were XDR/DTR, all isolated from medicine, except for one DTR strain isolated in the ICU. These findings show a worrying situation due to the likely presence of high-risk clones in this unit, as well as poor surveillance of multidrug-resistant strains, coupled with few treatment options for these infections, including ceftolozane/tazobactam, ceftazidime/avibactam, or imipenem/relebactam, which are not active against these strains ^18^^,^^19^.

In this study, we report high diversity of REP-PCR profiles, mostly found in the medicine ward, with unique profiles and high clonal diversity. These findings coincide with those reported by Tomas da Costa *et al*. ^20^, who, after using PFGE, found polyclonality in carbapenemase-producing *P. aeruginosa* from 12 Chilean hospitals.

Similar results were reported by Rodrigues *et al*. ^7^ after genotyping *P. aeruginosa* strains using MLST and automated REP-PCR in a hospital in the Amazon region of Brazil. In the same context, Oliver *et al*. ^18^ and Del Barrio-Tofiño *et al*. ^21^ reported that *P. aeruginosa* had a non-clonal epidemic population structure, composed of a limited number of widespread clones, which are selected from a large number of rare and unrelated genotypes that recombine at high frequency, and that global high-risk XDR/DTR clones are disseminated in hospitals worldwide.

In Peru, there are few studies on clonality of carbapenemase-producing *P. aeruginosa* in healthcare centers, possibly due to the lack of practical and economical methodologies that allow for the clonal relationship of isolates to be determined in a shorter period of time. Therefore, one of the strengths of this study is that it demonstrates that the REP-PCR technique may be an alternative for hospitals. Some of the most important limitations of this study were the failure to sequence the entire genome to determine the allelic variants of MBLs and the identification of high-risk clones, as well as the small number of evaluated isolates. All of these issues will be addressed in future studies.

In conclusion, we report the polyclonality of MBL-producing *P. aeruginosa* circulating in a tertiary hospital in Lima using the REP-PCR technique, which has proven to be a practical and economical alternative in routine epidemiological surveillance.
