# Supplementary material for: Clonal diversity of carbapenemase-producing Pseudomonas aeruginosa isolated from clinical samples in a third level hospital in Peru
Source: Rev Peru Med Exp Salud Publica. 2025 Feb 17;42(1):76–81. doi: 10.17843/rpmesp.2025.421.13818 (PMC12176022; doi:10.17843/rpmesp.2025.421.13818)
Supplement: Translation

# Translation: Diversidad clonal de *Pseudomonas aeruginosa* productoras de carbapenemasas aisladas de muestras clínicas en un hospital de tercer nivel de Perú

**Authors:** Gina Salvador-Lujan, Liz Erika Cruz-Pio, Hedersson Calla, Damaris Rivera-Asencios, Luis Solís-Cayo, Ruth García-de-la-Guarda

**Affiliations:** 1 Laboratorio de Microbiología del Hospital Militar Central «Crl. Luis Arias Schreiber», Lima, Perú.; 2 Laboratorio de Ecología Microbiana, Facultad de Ciencias Biológicas, Universidad Nacional Mayor de San Marcos, Lima, Perú.; 3 Laboratorio de Microbiología Molecular y Biotecnología, Facultad de Ciencias Biológicas, Universidad Nacional Mayor de San Marcos, Lima, Perú.

**Keywords:** Pseudomonas aeruginosa, Metalo-β-lactamasas, Tipificación Molecular, Carbapenemasas

## Abstract

*Pseudomonas aeruginosa* es un patógeno oportunista relacionado con infecciones de atención en salud, tiene elevados niveles de resistencia a los antimicrobianos y se asocia a brotes hospitalarios. La detección temprana de brotes es un problema en la rutina de los hospitales, por lo que se investigó la relación clonal de *P. aeruginosa* productora de carbapenemasas en un hospital de tercer nivel en Lima, Perú. Se recolectaron 24 cepas de *P. aeruginosa* productoras de metalo β-lactamasas aisladas de pacientes hospitalizados y se determinó la relación clonal utilizando la técnica REP-PCR. Los perfiles de bandas REP-PCR fueron normalizados, analizados y combinados mediante el programa BioNumerics versión 7,6. La identificación molecular mostró 19 perfiles diferentes y se encontraron cuatro grupos clonales. Se determinó la policlonalidad entre los aislados. No se encontró diseminación clonal entre las cepas de *P. aeruginosa* productoras de metalo-β-lactamasas que circulan en el hospital.

## INTRODUCCIÓN

*Pseudomonas aeruginosa* es considerada un patógeno oportunista de gran relevancia clínica por la alta morbimortalidad asociada con las infecciones relacionadas con la atención en salud ^1^^,^^2^. En 2020, el Centers for Disease Control and Prevention (CDC) informó 28 800 casos y 2500 muertes por *P. aeruginosa* resistentes a múltiples fármacos (MDR) en pacientes hospitalizados, cifras que se incrementaron durante la pandemia de COVID-19 ^3^.

El tratamiento de las infecciones por *P. aeruginosa* es cada vez más limitado debido a su elevada capacidad de desarrollar resistencia a los antimicrobianos por mecanismos de expresión constitutiva de cefalosporinasas derivadas de *Pseudomonas* (PDC, por sus siglas en ingles), modificaciones de las bombas de eflujo, baja permeabilidad de la membrana externa, producción de betalactamasas y resistencia adaptativa por condiciones de estrés, lo que la convierte en un importante problema de salud pública ^1^^,^^2^^,^^4^.

En *P. aeruginosa* la resistencia a los antibióticos carbapenémicos (último recurso para infecciones por bacterias MDR) se debe principalmente a la disminución de OprD, sobreexpresión de bombas de eflujo, sobreproducción de PDC y/o la combinación de estos mecanismos, sin embargo, las carbapenemasas codificadas en plásmidos e integrones adquiridos por transferencia horizontal son de gran relevancia, destacando las metalo-β-lactamasas (MBL) ^(^^2^^,^^4^. Las MBL presentan diversas variantes que originan diseminación nosocomial de clones de alto riesgo y de cepas MDR, extremadamente resistentes (XDR), panrresistentes (PDR) o difíciles de tratar (DTR), por ello es importante la vigilancia de la diseminación de clones para su detección temprana y evitar brotes ^1^^,^^4^. Entre las técnicas de referencia utilizadas para la tipificación molecular está la electroforesis en campo pulsado (PFGE, por sus siglas en inglés) por su elevado poder de discriminación, pero entre sus limitaciones está el costo elevado del equipo, su laboriosidad, la dificultad de diferenciar los pulsotipos con las bandas de ADN próximas y el tiempo requerido para analizar los pulsotipos ^(^^5^^,^^6^.

Otras técnicas utilizadas son las secuencias de ADN repetidas (REP-PCR), son económicas, menos laboriosas, rápidas y suelen generar patrones de bandas relativamente más fáciles de interpretar que las obtenidas por PFGE, aunque el poder de discriminación de esta técnica de PCR puede ser inferior a los obtenidos por PFGE ^(^^5^. En la técnica REP-PCR (*Repetitive Extragenic Palindromic*) se amplifican secuencias repetidas con elementos palindrómicos entre 35-40 pb, originando una gran variabilidad de perfiles de bandas de ADN en función al número de secuencias repetitivas y la distancia entre dichas secuencias, por ello es empleada con éxito para fines epidemiológicos y taxonómicos, comercializada en presentaciones semiautomatizadas como Diversilab (bioMérieux) ^7^.

 En el 2016, en el Hospital Militar Central, se aislaron e identificaron 24 cepas de *P. aeruginosa* altamente resistentes a los antibióticos y productoras de MBL, procedentes de diferentes unidades de hospitalización ^(^^8^. El objetivo del presente estudio fue investigar la relación genética (clonalidad) entre las cepas de *P. aeruginosa* MBL utilizando la técnica REP-PCR.

**MENSAJE CLAVE**

Motivación para el estudio. El aislamiento de *Pseudomonas aeruginosa* productora de carbapenemasas en diferentes salas de hospitalización de un hospital de tercer nivel de atención, motivó la identificación de la clonalidad de los aislados y determinar si correspondían a un brote intrahospitalario.

Principales hallazgos. La técnica REP-PCR agrupó a las 24 cepas de *P. aeruginosa* productoras de metalo-β-lactamasas aisladas de pacientes en diferentes salas de hospitalización, en 19 perfiles. La mayor diversidad clonal se observó en la unidad hospitalaria de medicina.

Implicancias para la salud pública. La tipificación molecular por REP-PCR podría ser una alternativa práctica y rápida en la vigilancia y control de brotes hospitalarios.

## EL ESTUDIO

Se llevó a cabo un estudio descriptivo de corte transversal. Se emplearon 24 cepas de *P. aeruginosa* resistentes a los carbapenemes por MBL, procedentes de secreciones respiratorias, heridas, orina y sangre, aisladas de enero a setiembre del 2016. La susceptibilidad a los antimicrobianos: imipenem, meropenem, ceftazidime, cefepime, piperacilina tazobactam, ciprofloxacina, gentamicina y amikacina, se realizó por disco difusión según el *Clinical and Laboratory Standars Institute* (CLSI) ^9^ y la colistina por elución de disco en caldo (CLSI). La definición de MDR, XDR se realizó según Magiorakos *et al*. ^10^ y las de DTR según Kadri *et al*. ^11^.

Las cepas fueron reactivadas y sembradas en caldo Luria-Bertani (Difco, le Pont de Claix, France) pH 7,0 e incubadas a 37 ºC por 12-14 horas y para extraer el ADN genómico se utilizó el Wizard® Genomic DNA Purification Kit (Promega, Madison, Wisconsin, USA) siguiendo las instrucciones del fabricante. El ADN genómico extraído y purificado fue cuantificado en un NanoDrop One (ThermoFisher Scientific, Madison, USA).

Para el análisis molecular por REP-PCR, se empleó el termociclador Applied Biosystems ProFlex PCR System (ThermoFisher Scientific, Madison, USA) y los cebadores REP-1-F (5’-III GCGCCGICATCAGGC-3’) y REP-2-R (5’-ACGTCTTATCAGGCCTAC-3’) previamente descritos por Versalovic *et al*. ^12^ con las siguientes modificaciones en las condiciones de amplificación: una desnaturalización inicial (95 °C por 2 minutos), seguido de 30 ciclos de desnaturalización (95 ºC por 30 segundos), hibridación (60 °C por 45 segundos), extensión (70 ºC por 1 minuto) y un ciclo final de extensión a 70 °C por 10 minutos. Cada reacción de PCR se llevó a cabo en un volumen total de 25 µL, se utilizó la ADN polimerasa KOD Hot Start DNA Polymerase (Novagen Millipore, Billerica, MA USA) y como molde se utilizó 1 µL del ADN genómico purificado. Simultáneamente, se utilizó agua para biología molecular como control negativo y como controles de amplificación se utilizaron *P. aeruginosa* ATCC 27853 y *Escherichia coli* ATCC 25922.

Los productos de amplificación fueron separados en gel de agarosa (Sigma-Aldrich, St. Louis, MO, USA) al 1,5% (p/v). Y para la electroforesis se utilizó el tampón TAE 50mM (Tris acetato EDTA 50 mM, pH 8,5) a 50 V por 2,5 horas. El tamaño de los fragmentos se estimó por comparación con el marcador de peso molecular GeneRuler 1 kb DNA Ladder (ThermoFisher Scientific, Madison, USA) y las bandas fueron visualizadas con GelRed® Nucleic Acid Gel Stain (Biotium, San Francisco Bay Area, USA) a 20X y digitalizadas con un sistema de documentación.

Los perfiles de bandas REP-PCR fueron normalizados, analizados y combinados mediante el programa BioNumerics versión 7,6 (Applied Maths, Kortrijk, Belgium). Para determinar el umbral de discriminación se realizó según la metodología Rodas *et al*. ^13^ y Cruz-Pio *et al*^14^.

La investigación fue aprobada por el Comité de Ética del Hospital Militar Central (Oficios: N°285AA-11/a/01.01 y N°1193AA-11/8/HMC/DADCI). Los datos de identificación de las cepas aisladas se trabajaron de forma confidencial.

## HALLAZGOS

El 54,2% de las de *P. aeruginosa* MBL se colectó de secreciones respiratorias, 29,2% de heridas, 12,5% de orinas y 4,1% de sangre. Se identificaron los genes *bla*_IMP_ (95,8%) y *bla*_VIM_ (4,2%). Según la unidad de hospitalización, se distribuyeron en un 79,2% en medicina, 12,5% en unidad de cuidados intensivos (UCI) y el 4,2% en cirugía y neumología ^(^^8^ (tabla 1).

**Tabla 1:** *Pseudomonas aeruginosa* productora de metalo-β-lactamasas y su relación con las muestras de pacientes hospitalizados.

Código	Cepa			Datos del paciente		Unidad hospitalaria
	Fecha de aislamiento	Muestra	**Gen *bla***	Sexo	Edad	
334	29-04-2016	SR	IMP	M	82	MED
414	15-16-2016	SR	IMP	M	52	MED
751	08-09-2016	SR	IMP	M	75	MED
809	23-09-2016	SR	IMP	M	72	MED
28	25-01-2016	SR	IMP	M	69	MED
383	04-06-2016	SR	IMP	M	97	MED
869	05-10-2016	SR	IMP	M	32	MED
814	24-09-2016	SR	IMP	F	59	MED
925	17-10-2016	SR	IMP	M	76	MED
408	14-06-2016	H	IMP	M	64	MED
707	02-09-2016	H	IMP	M	64	MED
147	12-02-2016	H	IMP	M	85	MED
862	01-10-2016	H	IMP	M	54	MED
912	13-10-2016	H	IMP	F	55	MED
47	30-01-2016	H	IMP	M	26	MED
792	20-09-2016	H	IMP	M	66	MED
369	06-05-2016	O	IMP	F	82	MED
347	06-03-2016	O	VIM	M	63	MED
382	08-09-2016	HEMO	IMP	M	37	MED
539	24-07-2016	SR	IMP	M	89	UCI
769	14-07-2016	SR	IMP	M	56	UCI
778	15-07-2016	SR	IMP	M	87	UCI
333	29-04-2016	SR	IMP	M	20	NEU
947	24-10-2016	O	IMP	M	90	CIR

MED: medicina; UCI: unidad de cuidados intensivos; NEU: neumología; CIR: cirugía; H: herida; SR: secreción respiratoria; O: orina; HEMO: sangre, F: femenino; M: masculino; *bla*_IMP_: gen Imipenemasa; *bla*_VIM_: gen Verona Integron-encoded Metallo β- lactamase.

Todas fueron resistentes a meropenem, imipenem y ceftazidime, 95,8% a cefepime, 87,5% a gentamicina y ciprofloxacina, 83,3% a amikacina, 33,3% a piperacilina tazobactam, 12,5% a aztreonam ^(^^8^^).^ Todas fueron susceptibles a colistina y, según el perfil de resistencia, el 70,8% (17/24) fueron MDR y el 29,2% (7/24) XDR/DTR. No se detectaron perfiles PDR (Figura 1).

**Figura 1:** Dendrograma que muestra la relación clonal de 24 cepas de *Pseudomonas aeruginosa* productoras de metalo-β-lactamasas, utilizando el coeficiente de correlación cofenético de Pearson y el método de agrupamiento de la media aritmética ponderada UPGMA (*Unweighted-Pair Group Method with Aritmetic Mean*). La línea pun-teada representa el 96% de similaridad.

MDR: resistencia a múltiples fármacos; XDR: extremadamente resistente; DTR: difícil de tratar; ATCC: American Type Culture Collection.

Tras la tipificación, las 24 cepas de *P. aeruginosa* MBL se agruparon en 19 perfiles REP-PCR con un nivel de similitud de 96% y con un coeficiente de correlación de Pearson del 90% (optimizado al 1%, tolerancia al 2,5%) es decir estos 19 perfiles de *P. aeruginosa* MBL son genéticamente distintos y de aquí en adelante se considerarán como cepas distintas (figura 1). La reproducibilidad de los perfiles obtenidos fue de 98% y fue el resultado del valor promedio de la similaridad de tres cepas analizadas por duplicado.

De los perfiles REP-PCR obtenidos, el 78,9% (15/19) tuvieron como representante sólo una cepa y presentaron perfiles REP-PCR únicos, es decir, sin ninguna relación genética entre ellas; de estos, el 73,3% (11/15) correspondió a medicina. En UCI, se encontraron los perfiles VIII, XVI y XIX, representando el 15,8% de los perfiles únicos. En neumología, se encontró el perfil XV y en cirugía el perfil IV, representando en ambos casos el 5,3%.

Se encontraron cuatro grupos clonales, el perfil IV (cepas 925 y 947) en medicina y cirugía. El perfil XI (cepas 334, 862 y 814), perfil XIV (cepas 28 y 792) y el perfil XVIII (cepas 408 y 414). Estos tres últimos grupos clonales fueron encontrados en medicina.

Según el perfil de resistencia, en medicina se aislaron el 82,3% (17/24) de las MDR y el 71,4% (5/7) DTR, en UCI se aisló el 28,6% (2/7) de las cepas DTR. La unidad que presentó mayor cantidad de cepas y diversidad de perfiles fue medicina, con 11 perfiles únicos y 3 grupos clonales.

## DISCUSIÓN

En este estudio reportamos la alta diversidad clonal de *P. aeruginosa* MBL con perfiles de resistencia MDR y XDR/DTR circulando principalmente en la unidad hospitalaria de medicina de un hospital de tercer nivel de atención en Lima.

*P. aeruginosa* resistente a carbapenémicos es uno de los principales microorganismos causantes de infecciones asociadas a la atención en salud, por ello, la Organización Mundial de la Salud lo sigue considerando en la lista de los patógenos bacterianos de alta prioridad en investigación, desarrollo y búsqueda de nuevas estrategias de prevención y control de las infecciones ^(^^15^. La detección temprana de *P. aeruginosa* productora de carbapenemasas en pacientes colonizados o infectados es de suma importancia en los centros de atención de salud, para evitar su propagación y prevenir los brotes hospitalarios.

A nivel mundial, se han reportado infecciones por *P. aeruginosa* resistentes a carbapenémicos en pacientes hospitalizados, originando brotes en diferentes unidades de hospitalización, principalmente en UCI ^16^^,^^17^. Durante el 2016, en el Hospital Militar Central se registró un incremento de aislamientos de *P. aeruginosa* resistentes a los carbapenemes por MBL, identificándose el gen *bla*_IMP_ y *bla*_VIM_^(^^8^. El 69,2% de las secreciones respiratorias se aislaron de medicina, estos hallazgos coinciden con los reportados por Vega *et al*. ^2^ quienes reportaron el 51,3% de sus aislamientos de *P. aeruginosa* MBL de infecciones respiratorias en un centro asistencial académico de Miami, Florida.

Actualmente, la diseminación de clones de alto riesgo de *P. aeruginosa* MDR/XDR es un problema de salud pública, poniendo en riesgo la actual terapia antimicrobiana y aumentando la morbimortalidad ^(^^18^. En España, Sastre-Femenia *et al*. ^(^^19^ evidenciaron una disminución de los perfiles XDR, DTR de resistencia de *P. aeruginosa* y una ligera disminución de la prevalencia de betalactamasas de espectro extendido y carbapenemasas en el 2022; sin embargo, hubo un aumento de cepas XDR productoras de carbapenemasas asociadas al clon ST235 de alto riesgo hipervirulento. En nuestro estudio el 70,8% de *P. aeruginosa* MBL fueron MDR y el 29% XDR/DTR, todas aisladas de medicina excepto una cepa DTR aislada en UCI. Estos hallazgos muestran una situación preocupante por la probable presencia de clones de alto riesgo en esta unidad, así como la deficiente vigilancia de las cepas multirresistentes, aunado a las pocas opciones de tratamiento para estas infecciones incluyendo al ceftolozano/tazobactam, ceftazidima/avibactam o imipenem/relebactam que no son activos frente a estas cepas ^(^^18^^,^^19^.

En este estudio reportamos, la alta diversidad de perfiles REP-PCR en su mayoría encontrados en medicina con perfiles únicos y alta diversidad clonal. Estos hallazgos coinciden con lo reportado por Tomas da Costa *et al*. ^(^^20^, quienes tras utilizar PFGE observaron la policlonalidad en *P. aeruginosa* productoras de carbapenemasas procedentes de 12 hospitales chilenos.

Similares resultados fueron reportados por Rodrigues *et al.*^7^ tras genotipificar cepas de *P. aeruginosa* utilizando MLST y REP-PCR automatizado en un hospital de la Amazonía en Brasil. En el mismo contexto Oliver *et al*. ^18^ y Del Barrio-Tofiño *et al*. ^21^ reportan que *P. aeruginosa* tiene una estructura de población epidémica no clonal, compuesta por un número limitado de clones generalizados, que se seleccionan de una gran cantidad de genotipos raros y no relacionados, que se recombinan con alta frecuencia y los clones globales de alto riesgo XDR/DTR están diseminados en los hospitales de todo el mundo.

En el Perú, son pocos los estudios de clonalidad en *P. aeruginosa* productoras de carbapenemasas en los centros asistenciales de salud, posiblemente por la falta de metodologías prácticas y económicas que permitan conocer, en menor tiempo, la relación clonal de los aislados, por ello una fortaleza de este estudio es evidenciar que la técnica REP-PCR puede representar una alternativa en los hospitales. Entre las limitaciones identificamos la no secuenciación del genoma completo para determinar las variantes alélicas de las MBL y la identificación de clones de alto riesgo, así como el número reducido de aislados evaluados, todas ellas serán abordadas en próximos estudios.

En conclusión, reportamos la policlonalidad de *P. aeruginosa* productoras de MBL que circulan en un hospital de tercer nivel de Lima utilizando la técnica de REP-PCR, que ha demostrado ser una alternativa práctica y económica en la rutina de la vigilancia epidemiológica.
